# SHP-1/STAT3-Signaling-Axis-Regulated Coupling between BECN1 and SLC7A11 Contributes to Sorafenib-Induced Ferroptosis in Hepatocellular Carcinoma

**DOI:** 10.3390/ijms231911092

**Published:** 2022-09-21

**Authors:** Chao-Yuan Huang, Li-Ju Chen, Grace Chen, Tzu-I Chao, Cheng-Yi Wang

**Affiliations:** 1 Division of Radiation Oncology, Department of Oncology, National Taiwan University Hospital, College of Medicine, National Taiwan University, Taipei 100229, Taiwan; 2 School of Medicine, College of Medicine, Taipei Medical University, Taipei 110301, Taiwan; 3 SupremeCure Pharma Inc., Taipei 106047, Taiwan; 4 Department of Internal Medicine, Cardinal Tien Hospital and School of Medicine, College of Medicine, Fu Jen Catholic University, New Taipei City 231009, Taiwan

**Keywords:** ferroptosis, STAT3, BECN1, SLC7A11, HCC

## Abstract

Ferroptosis is a type of iron-dependent cell death pertaining to an excess of lipid peroxidation. It has been suggested that sorafenib—an anti-angiogenic medication for hepatocellular carcinoma (HCC)—induces ferroptosis, but the underlying mechanism for this remains largely unknown. We employed siRNA-mediated gene silencing to investigate the role of Src homology region 2 domain-containing phosphatase-1 (SHP-1), following sorafenib treatment, in cystine/glutamate-antiporter-system-Xc^−^-regulated cystine uptake. Co-immunoprecipitation was also performed to examine the interactions between MCL1, beclin 1 (BECN1), and solute carrier family 7 member 11 (SLC7A11), which functions as the catalytic subunit of system Xc^−^. The results of this study showed that sorafenib enhanced the activity of SHP-1, dephosphorylated STAT3, downregulated the expression of MCL1 and, consequently, reduced the association between MCL1 and BECN1. In contrast, increased binding between BECN1 and SLC7A11 was observed following sorafenib treatment. The elevated interaction between BECN1 and SLC7A11 inhibited the activity of system Xc^−^, whereas BECN1 silencing restored cystine intake and protected cells from ferroptosis. Notably, ectopic expression of MCL1 uncoupled BECN1 from SLC7A11 and rescued cell viability by attenuating lipid peroxidation. The results revealed that ferroptosis could be induced in HCC via SHP-1/STAT3-mediated downregulation of MCL1 and subsequent inhibition of SLC7A11 by increased BECN1 binding.

## 1. Introduction

Liver cancer ranks as the sixth most commonly occurring type of malignancy, and it is also the third leading cause of cancer-associated death [1]. Among all types of liver cancer, hepatocellular carcinoma (HCC) is the most dominant, representing approximately 85–90% of primary liver cancers. From the approval of sorafenib [2]—the first systemic treatment—to the development of an immunotherapy combination (atezolizumab plus bevacizumab [3]), substantial improvements in the treatment of advanced HCC have been made over the last few decades. However, approximately 70% of patients still do not respond to current treatment options.

Ferroptosis is a lipid-peroxidation-driven and iron-dependent form of regulated cell death [4]. Iron serves as a catalytic subunit for enzymes involved in mediating ferroptosis, and it is also required in the non-enzymatic Fenton reaction for the generation of reactive oxygen species (ROS) [5]. An excess of ROS can attack lipid membranes, leading to uncontrolled lipid peroxidation, which compromises the integrity of cellular membranes and results in ferroptotic cell death. Ferroptosis can be induced via external stimulation by targeting the cystine/glutamate antiporter system Xc^−^, which comprises the anchoring solute carrier family 3 member 2 (SLC3A2) subunit and the catalytic solute carrier family 7 member 11 (SLC7A11) subunit [6]. The antiporter system Xc^−^ exports glutamate and imports cystine at an equal ratio. The imported cystine is further reduced to cysteine for the production of intracellular glutathione (GSH). The antioxidant GSH serves as a reducing substrate for glutathione peroxidase 4 (GPX4) to reduce lipid hydroperoxides and lipid ROS. Hence, the inhibition of system Xc^−^ leads to a redox imbalance, the accumulation of lipid peroxidation, and ferroptosis. Ferroptosis can also be triggered by internal signals, through the inhibition of antioxidant defense molecules such as GPX4. GPX4 protects cells from ferroptosis by converting lipid peroxides to less toxic lipid alcohols, while catalyzing the production of oxidized glutathione (GSSG). GPX4 inhibition therefore impairs cellular defenses against lipid peroxidation and contributes to ferroptosis. Considering that tumor cells generally have a higher demand for iron to support their proliferation [7,8], and that the liver is the primary organ for the storage of excess iron, the concept of inducing ferroptosis and triggering HCC cell death has drawn much attention.

Sorafenib is an oral kinase inhibitor approved for the treatment of advanced HCC [2,9]. Sorafenib was developed to inhibit the activity of vascular endothelial growth factor receptors, rapidly accelerated fibrosarcoma kinases, and platelet-derived growth factor receptors [10]; this, in turn, suppresses cancer cell proliferation, as well as the angiogenesis required for tumor growth. Furthermore, sorafenib may also combat HCC via other mechanisms, such as promoting pyroptosis in macrophages and facilitating natural-killer-cell-triggered anti-HCC activities [11]. Louandre et el. mentioned that sorafenib induces iron-dependent ferroptotic cell death in HCC [12], but the detailed molecular machinery through which sorafenib triggers ferroptosis in HCC remains largely unclear.

Signal transducer and activator of transcription 3 (STAT3) is a transcription factor that regulates the expression of molecules involved in inflammation, survival, proliferation, and differentiation [13]. It has been suggested that propofol triggers ferroptosis by reducing the expression of STAT3 in colorectal cancer cells, and that ectopic expression of STAT3 alleviates ferroptosis [14]. Hence, STAT3 may play a role in the negative regulation of ferroptosis. Previous studies found that sorafenib enhances the activity of Src homology region 2 domain-containing phosphatase-1 (SHP-1) and dephosphorylates STAT3 [15]. We therefore wondered whether the activation of SHP-1 and inhibition of STAT3 by sorafenib would have profound effects on sorafenib-induced ferroptosis in HCC.

In this study, we aimed to identify key molecules and dissect the molecular interplays that contribute to sorafenib-induced ferroptosis in HCC. The expression of SHP-1 and beclin 1 (BECN1) was silenced to determine their roles in sorafenib-triggered ferroptosis in HCC. Co-immunoprecipitation experiments were conducted to investigate the molecular interactions that gave rise to ferroptosis following sorafenib treatment. Our research was conducted using clinically available sorafenib which, unlike some early-stage ferroptosis-inducing agents that have been limited by their instability, indicates that HCC may be vulnerable to ferroptotic cell death. The aforementioned concept underlines the potential of exploiting ferroptosis-inducing agents to combat HCC or to sensitize current treatment options against HCC.

## 2. Results

### 2.1. Sorafenib Treatment Enhances Lipid Reactive Oxygen Species (ROS) Levels and Induces Ferroptotic Cell Death in Hepatocellular Carcinoma (HCC) Cells

**Figure 1:**
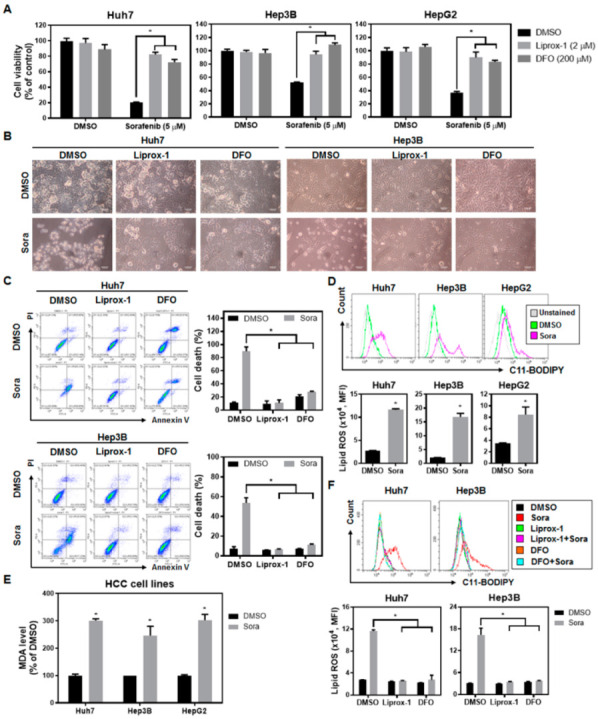
Sorafenib induces ferroptosis in hepatocellular carcinoma (HCC) cells: (**A**) Huh7, Hep3B, and HepG2 cells cultured in complete Dulbecco’s modified Eagle medium (DMEM) medium were treated with sorafenib with or without ferroptosis inhibitors (Liprox-1 and DFO) for 16 h, and cell viability was quantified by a 3-[4,5-dimethylthiazol-2-yl]-2,5-diphenyltetrazolium bromide (MTT) assay. (**B**) Images of cell morphology for HCC cells treated with dimethyl sulfoxide (DMSO) (0.3%) or sorafenib (5 µM), in the presence or absence of the indicated ferroptosis inhibitors, for 16 h. Scale bar = 100 µm. (**C**) Cell death (PI-positive population, %) evaluation was performed using an Annexin V/PI Assay Kit. After exposure to sorafenib with or without ferroptosis inhibitors for 16 h, cells were subjected to flow cytometry analyses. (**D**) The extent of lipid ROS accumulation was determined in three HCC cells lines treated with sorafenib (5 µM) for 16 h using C11-BODIPY. (**E**) Sorafenib treatment significantly enhanced the amount of malondialdehyde (MDA) in HCC cells. Cells were treated with sorafenib (2.5 µM for Huh7 and HepG2 cells; 5 µM for Hep3B cells) for 16 h and then the level of lipid peroxidation was determined. (**F**) The use of the ferroptosis inhibitors Liprox-1 and DFO reduced lipid ROS levels in HCC cells treated with sorafenib. Columns: mean; bars: SD (n ≥ 3). Statistical significance was evaluated using the Mann–Whitney U test (*, *p* < 0.05).

The HCC cell lines Huh7, Hep3B, and HepG2, treated with 5 µM of sorafenib [16], resulted in cell death, while the use of the ferroptosis inhibitors liproxstatin-1 (Liprox-1) [17] and deferoxamine mesylate (DFO) [18] significantly rescued sorafenib-induced cell death (Figure 1A). Changes in cell morphology, including the rounding-up of cells and their subsequent death, were observed in sorafenib-treated HCC cells (Figure 1B). Interestingly, the morphological changes induced by sorafenib could also be reversed by the application of ferroptosis inhibitors (Figure 1B). The results acquired via Annexin V probes and propidium iodide (PI) staining further corroborated the fact that ferroptosis inhibitors significantly protected HCC cells from sorafenib-induced cell death (Figure 1C). These results indicate that ferroptosis-associated cell death may contribute to a large portion of HCC cell death triggered by sorafenib.

Since lipid-ROS-triggered lipid peroxidation is a critical event driving ferroptosis, we examined the correlation between sorafenib treatment and the levels of lipid ROS within HCC cells. Treatment of HCC cells with sorafenib increased the levels of both lipid ROS (Figure 1D) and malondialdehyde (MDA) (Figure 1E), which is a final product of lipid peroxidation. Co-incubation of sorafenib with two types of ferroptosis inhibitors—the scavenger of lipid ROS Liprox-1 and the iron chelator DFO—significantly reduced the accumulation of lipid ROS (Figure 1F). These findings imply that sorafenib treatment may lead to increased lipid ROS inside cells, leading to an excess of lipid peroxidation and the triggering of ferroptotic cell death in HCC cells.

### 2.2. Sorafenib Inhibits System-Xc^−^-Mediated Cystine Intake and Enhances Lipid ROS Levels within HCC Cells, Contributing to Ferroptotic Cell Death

**Figure 2:**
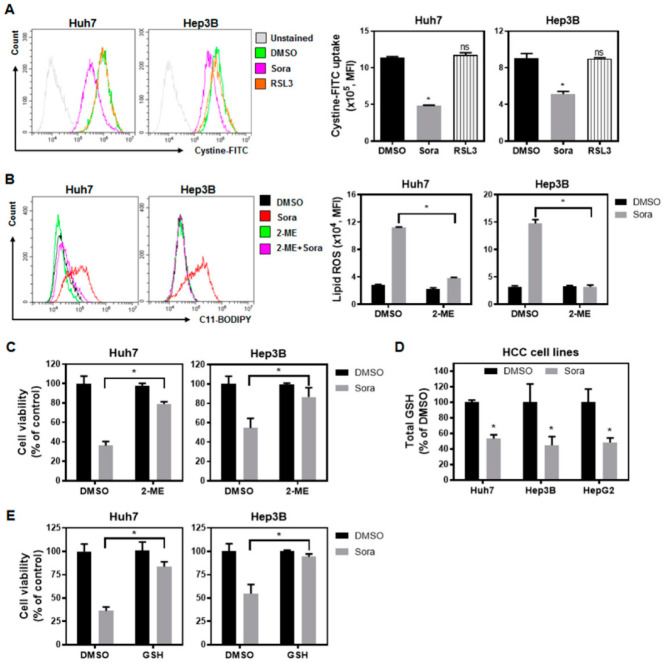
Sorafenib treatment inhibits system-Xc^−^-regulated cystine uptake and elevates lipid reactive oxygen species (ROS) levels, culminating in ferroptotic cell death: (**A**) Sorafenib inhibited cystine–FITC intake, whereas Ras-selective lethal small molecule 3 (RSL3)—a GPX4 inhibitor—did not. Huh7 cells and Hep3B cells were cultured in DMSO (0.1%), sorafenib (2.5 µM for Huh7 cells and 5 µM for Hep3B cells), or RSL3 (2.5 µM) for 16 h. After treatment, the cells were subjected to flow cytometry analyses. (**B**) The addition of β-Mercaptoethanol (2-ME) (50 µM) suppressed sorafenib (5 µM)-induced lipid ROS levels and (**C**) restored HCC cell viability. (**D**) Sorafenib treatment lowered the intracellular levels of total glutathione (GSH) in the three HCC cell lines. Cells were exposed to sorafenib (2.5 µM for Huh7 and HepG2 cells; 5 µM for Hep3B cells) for 16 h and then harvested for measurement of their GSH content. (**E**) The addition of reduced GSH (2 mM) to the medium containing 5 µM sorafenib decreased the cytotoxicity of sorafenib against Huh7 and Hep3B cells. MTT assays were conducted to determine the cell viability. Columns: mean; bars: SD (n ≥ 3). Statistical analysis was conducted by employing the Mann–Whitney U test (*, *p* < 0.05).

The cystine/glutamate antiporter system Xc^−^ imports cystine to synthesize GSH, which is a major antioxidant serving as a substrate for GPX4 [19], and protects cells from excessive lipid peroxidation. As sorafenib treatment enhanced the lipid ROS levels in HCC cells, we examined whether this increase in lipid ROS levels was the result of system Xc^−^ dysfunction following sorafenib treatment. As shown in Figure 2A, sorafenib inhibited the uptake of cystine–FITC, as evidenced by the reduced fluorescence intensity. On the other hand, Ras-selective lethal small molecule 3 (RSL3) [20]—a GPX4 inhibitor, but not an inhibitor of system Xc^−^—did not reduce cystine intake driven by system Xc^−^.

Cystine is transported through system Xc^−^ for its entry into cells, while cysteine is imported via the neutral amino acid transporter system. β-Mercaptoethanol (2-ME) reduces cystine to cysteine extracellularly, and can therefore be used to verify whether the suppressed activity of system Xc^−^ and the reduced cystine intake as a result of sorafenib treatment are critical for sorafenib-triggered ferroptosis. The levels of lipid ROS were significantly increased following sorafenib treatment (Figure 2B), and were accompanied by a decline in cells’ viability (Figure 2C). On the other hand, the application of 2-ME not only reduced the levels of lipid ROS in both Huh7 and Hep3B cells, but also restored cell viability (Figure 2B,C). Furthermore, we observed that sorafenib treatment reduced the intracellular total GSH levels (Figure 2D) in HCC cells—probably as a consequence of cystine depletion through sorafenib-mediated system Xc^−^ inhibition. Supplementing extracellular GSH alleviated sorafenib-triggered ferroptotic cell death (Figure 2E) in HCC cells. These findings suggest that sorafenib induces ferroptosis in HCC cells by inhibiting the activity of system Xc^−^ and reducing the cellular intake of cystine. Cystine deficiency further leads to decreased levels of intracellular GSH, and the subsequent accumulation of lipid peroxidation may contribute to sorafenib-induced HCC ferroptotic cell death.

### 2.3. The Src Homology Region 2 Domain-Containing Phosphatase-1 (SHP-1)/STAT3 Signaling Pathway Plays a Pivotal Role in Sorafenib-Induced Ferroptosis in HCC

**Figure 3:**
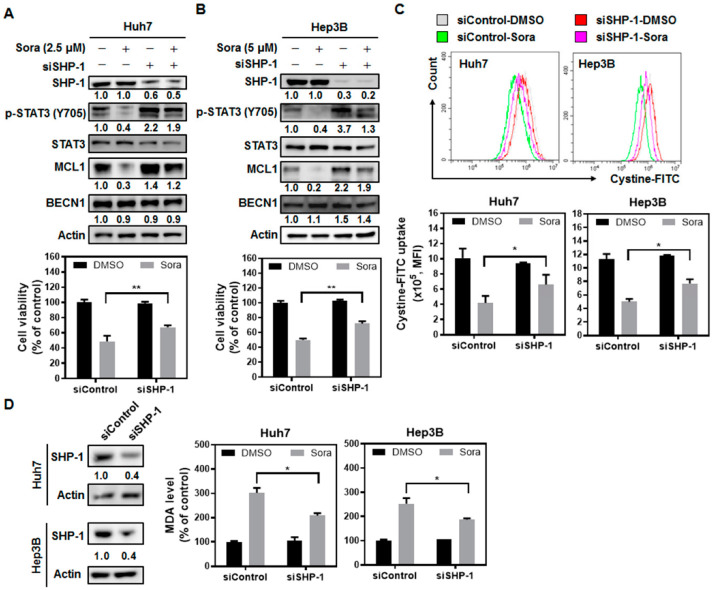
Src homology region 2 domain-containing phosphatase-1 (SHP-1) silencing rescues HCC cells from sorafenib-induced ferroptosis: (**A**) Huh7 and (**B**) Hep3B cells were transiently transfected with specific siRNA targeting SHP-1 (siSHP-1) overnight. Then, the cells were treated with 2.5 µM (Huh7 cells) or 5 µM (Hep3B cells) sorafenib for another 16 h. The expression of downstream molecules of SHP-1 was examined by immunoblotting (**upper panel**), and the cell viability was determined by MTT assay (**lower panel**). (**C**) After sorafenib treatment, the changes in cystine–FITC uptake and the extent of lipid peroxidation (**D**) were assessed via appropriate biochemical analyses. Scrambled control siRNA (siControl). Columns: mean; bars: SD (n ≥ 3). Statistical significance was assessed by employing the Mann–Whitney U test (*, *p* < 0.05; **, *p* < 0.01).

Previous studies have shown that sorafenib activates SHP-1 phosphatase and dephosphorylates STAT3 [15]. Lines of evidence also indicate that STAT3 may serve as a negative regulator of ferroptosis, as suppression of STAT3 activity induces ferroptosis in gastric cancer [17], while ectopic expression of STAT3 rescues colorectal cancer cells from propofol-triggered ferroptosis [14]. Hence, we investigated whether the SHP-1/STAT3 signaling pathway is involved in sorafenib-induced ferroptosis in HCC. Silencing SHP-1 expression led to enhanced STAT3 phosphorylation and relieved HCC cells from sorafenib-induced ferroptotic cell death (Figure 3A,B). Furthermore, SHP-1 silencing also restored the uptake of cystine (Figure 3C), which was inhibited by sorafenib. As a result, reduced lipid peroxidation was observed in SHP-1-silenced HCC cells following sorafenib treatment (Figure 3D). These data suggest that sorafenib-triggered ferroptosis in HCC relies, at least partially, on the activity of SHP-1.

**Figure 4:**
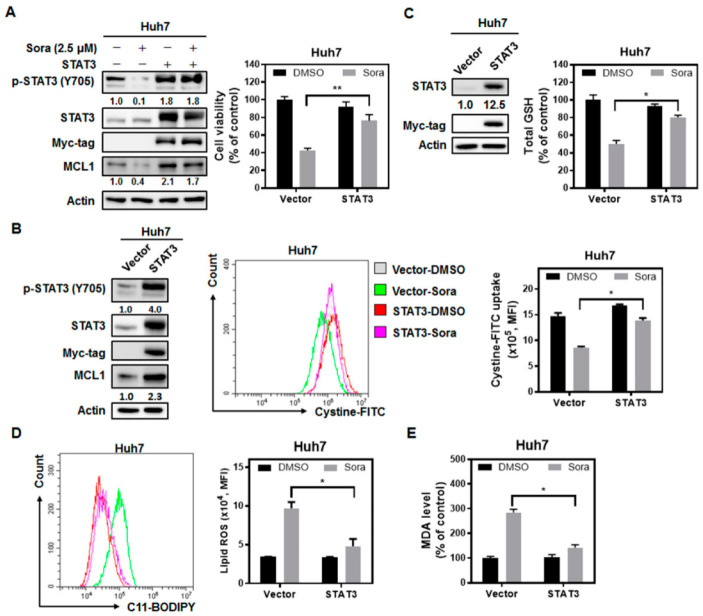
STAT3 plays a core role in mediating sorafenib-triggered ferroptosis in HCC cells: (**A**) Ectopic expression of STAT3 not only alleviated the inhibitory effects of sorafenib on MCL1 expression (**left panel**) and cell viability (**right panel**), but also (**B**) recovered cystine uptake, (**C**) restored GSH content, (**D**) reduced lipid ROS, and (**E**) lowered the levels of MDA. Columns: mean; bars: SD (n ≥ 3). Statistical significance was evaluated using the Mann–Whitney U test (*, *p* < 0.05; **, *p* < 0.01).

We next investigated the role of STAT3, which could be dephosphorylated and inactivated by SHP-1, in sorafenib-induced ferroptosis in HCC cells. Ectopically expressed STAT3 rescued cells from sorafenib-triggered ferroptotic cell death (Figure 4A), restored cystine uptake (Figure 4B) and total GSH levels (Figure 4C), decreased lipid ROS levels (Figure 4D), and reduced lipid peroxidation (Figure 4E) in HCC cells treated with sorafenib. The results highlight the role of SHP-1/STAT3 signaling in mediating sorafenib-induced ferroptosis in HCC.

### 2.4. Sorafenib Treatment Dissociates Beclin 1(BECN1) from MCL1, and the Released BECN1 Inhibits Solute Carrier Family 7 Member 11 (SLC7A11), Culminating in Sorafenib-Triggered Ferroptosis in HCC Cells

It has been reported that BECN1 promotes ferroptosis by directly binding with the catalytic component of system Xc^−^ (the SLC7A11 subunit) and blocking its activity [21]. It has also been indicated that MCL1, which is a downstream target of STAT3, binds with BECN1, preventing the formation of the core class III phosphoinositide 3-kinase complex during the initial step of autophagy [22]. Moreover, evidence has shown that the degradation of MCL1 frees BECN1 [23,24]. We hypothesized that sorafenib treatment dephosphorylated STAT3 and reduced MCL1 expression, promoting the binding of BECN1 with SLC7A11, and contributing to ferroptosis in HCC.

**Figure 5:**
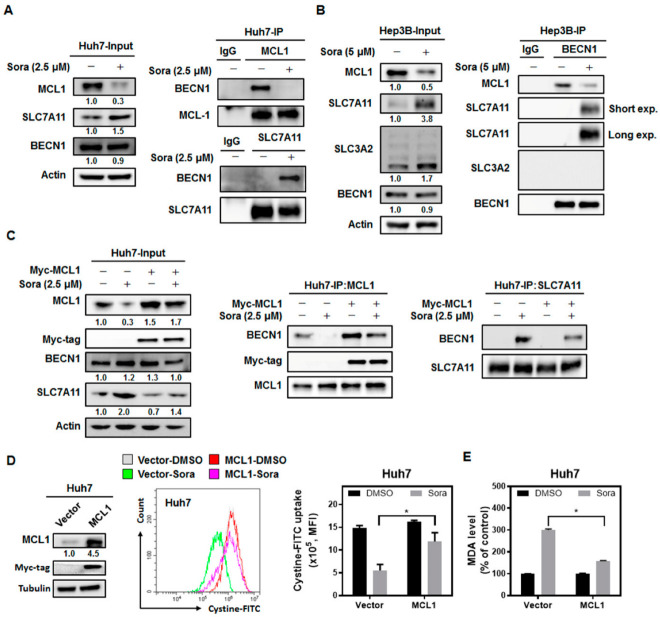
Sorafenib treatment reduces the interaction between MCL1 and beclin 1 (BECN1), while promoting the association between BECN1 and solute carrier family 7 member 11 (SLC7A11): (**A**) HCC cells were exposed to sorafenib (2.5 µM for Huh7 cells and 5 µM for Hep3B cells) for 16 h. MCL1- and SLC7A11-containing lysates were immunoprecipitated from sorafenib-treated Huh7 cells. Protein levels of MCL1, SLC7A11, BECN1, and actin were assayed with Western blots. (**B**) BECN1-containing lysates were immunoprecipitated from Hep3B cells incubated with 5 µM of sorafenib for 16 h. The expression of MCL1, SLC7A11, SLC3A2, and actin was further determined. (**C**) Ectopic expression of MCL1 diminished BECN1-to-SLC7A11 binding and (**D**) recovered the intake of cystine–FITC after 2.5 µM sorafenib treatment for 16 h. Columns: mean; bars: SD (n = 3). (**E**) Ectopic expression of MCL1 reduced MDA content following sorafenib treatment. Columns: mean; bars: SD (n ≥ 3). Statistical significance was analyzed using the Mann–Whitney U test (*, *p* <0.05).

Our results showed that sorafenib treatment downregulated the mRNA (Supplementary Figure S1A) and protein (Supplementary Figure S1B) expression of MCL1. Co-immunoprecipitation further revealed that sorafenib treatment diminished the binding of BECN1 to MCL1 (Figure 5A, upper right). Most importantly, by pulling down SLC7A11-containing lysates, increased binding of BECN1 to SLC7A11 was observed (Figure 5A, lower right). Enhanced binding between BECN1 and the SLC7A11 catalytic subunit of system Xc^−^ following sorafenib treatment was double confirmed by analyzing the immune complex captured by BECN1-specific antibodies (Figure 5B). On the other hand, SLC3A2—the anchoring subunit of system Xc^−^—was not detected in the BECN1-coimmunoprecipitated complex. Ectopic expression of MCL1 restrained the interaction between BECN1 and SLC7A11 (Figure 5C, right), restored cystine uptake (Figure 5D), reduced the levels of intracellular MDA (Figure 5E), and rescued cells from sorafenib-induced ferroptotic cell death (Supplementary Figure S2). These results imply that sorafenib treatment reduces the interaction between MCL1 and BECN1, so the released BECN1 binds to SLC7A11 and inhibits the transporter’s activity to import cystine.

**Figure 6:**
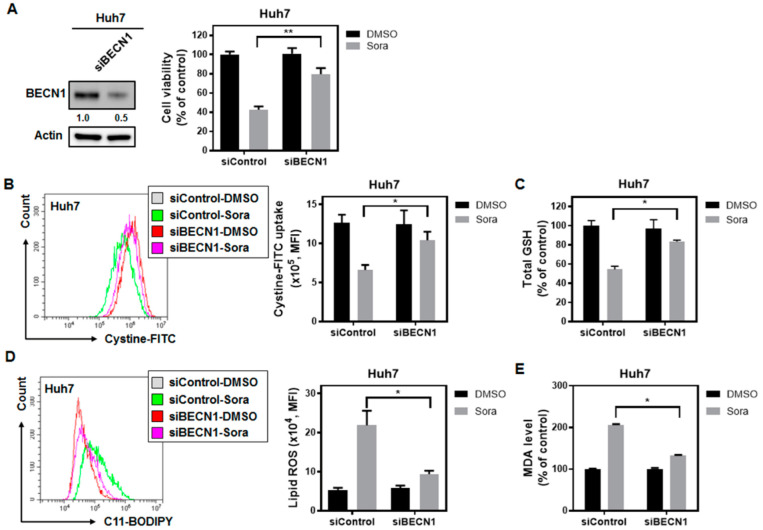
BECN1 silencing relieves sorafenib-triggered ferroptosis in HCC cells: (**A**) Silencing of BECN1 by siRNA in Huh7 cells alleviated sorafenib-induced ferroptotic cell death, (**B**) restored system Xc^−^’s transporter activity, (**C**) replenished intracellular total GSH, (**D**) reduced lipid ROS levels, and (**E**) restrained lipid peroxidation. Columns: mean; bars: SD (n ≥ 3). Statistical significance was evaluated using the Mann–Whitney U test (*, *p* < 0.05; **, *p* < 0.01).

As elevated binding was observed between BECN1 and SLC7A11, the role of BECN1 in sorafenib-induced ferroptosis was further verified. Silencing BECN1 significantly relieved sorafenib-triggered ferroptotic cell death in HCC cells (Figure 6A). It is noteworthy that the impaired activity of system Xc^−^ following sorafenib treatment was also restored in BECN1-silenced Huh7 cells (Figure 6B). Additionally, BECN1 knockdown not only rescued intracellular total GSH levels (Figure 6C), but also lowered the levels of lipid ROS (Figure 6D) and MDA (Figure 6E) in response to sorafenib treatment.

The aforementioned results highlight the importance of SLC7A11 in HCC and propelled us to examine its expression in patients suffering from HCC. Using the Tumor Immune Estimation Resource (TIMER) [25,26] to analyze the Cancer Genome Atlas (TCGA) database, we found that the expression of SLC7A11 was significantly higher (*p* < 0.001) in liver HCC (denoted as LIHC in the TIMER database) tumors (Supplementary Figure S3A) compared with their adjacent normal tissues. It is also notable that higher SLC7A11 expression was associated with significantly poorer survival (Supplementary Figure S3B; *p* < 0.01) for liver HCC patients (370 cases, 130 dying) based on the analysis using the TIMER database.

### 2.5. Sorafenib Induces Ferroptosis in the Huh7 Murine Xenograft Model

**Figure 7:**
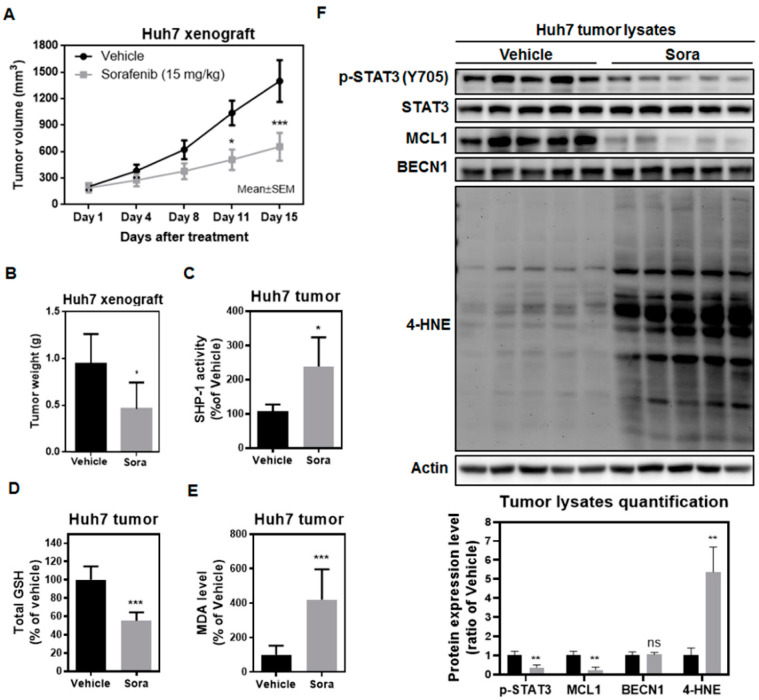
Sorafenib treatment enhances SHP-1 activity, inactivates STAT3, reduces MCL1 expression, and triggers ferroptosis in the Huh7 murine model: (**A**) Sorafenib inhibited tumor growth in Huh7 tumor-bearing mice. Sorafenib was orally administered five times per week, and the tumor volumes were measured twice weekly. Points: mean; bars: standard error of the mean (SEM) (n = 7). Statistical significance was evaluated by two-way ANOVA (*, *p* < 0.05; ***, *p* < 0.001). (**B**) At the end of the treatment, the Huh7 tumors were excised, and the tumor weights were recorded. (**C**) SHP-1 activities were measured using Huh7 tumor lysates from the mice treated with sorafenib or vehicle. (**D**) Total GSH levels and (**E**) MDA levels were evaluated using Huh7 tumors collected from the mice treated with sorafenib. (**F**) The protein expression levels (upper panel) and the quantification results (lower panel) of p-STAT3 (Y705), STAT3, MCL1, BECN1, and 4-HNE adducts were examined via Huh7 tumor lysates acquired from sorafenib-treated mice. Columns: mean; bars: SD (n ≤ 7). Statistical significance was determined by using the Mann–Whitney U test (*, *p* < 0.05; **, *p* < 0.01; ***, *p* < 0.001).

To investigate whether sorafenib also induces ferroptosis in vivo, we established the Huh7 xenograft model in mice. Sorafenib treatment reduced tumor volume (Figure 7A) and tumor weight (Figure 7B). Sorafenib also enhanced SHP-1 activity (Figure 7C), reduced the total GSH levels (Figure 7D), and increased the amounts of MDA in tumors (Figure 7E) collected from Huh7-bearing mice. Analyses using Huh7 tumor lysates demonstrated that there was reduced p-STAT3 and MCL1 expression (Figure 7F) following sorafenib treatment. The surge in 4-hydroxynonenal (4-HNE) adducts—a toxic byproduct of lipid peroxidation—also suggested that sorafenib induced ferroptosis in the Huh7 tumors (Figure 7F). Our in vivo data were consistent with what we observed in vitro—that sorafenib elicits its anti-HCC activities, at least partially, by triggering ferroptosis.

## 3. Discussion

There is growing recognition that the induction of ferroptosis represents another promising strategy for combating cancer. While sorafenib is known for showing anti-angiogenic activities, it could also suppress HCC growth by inhibiting the cystine/glutamate antiporter system Xc^−^ [27,28] and triggering ferroptosis. However, the underlying molecular machinery through which sorafenib inhibits the activity of system Xc^−^ is largely undetermined. We found that the SHP-1/STAT3 signaling axis serves as an integral component for mediating sorafenib-induced ferroptosis in HCC. SHP-1 has been shown to be a target of sorafenib [29], and its phosphatase activity increases following sorafenib treatment [15]. Silencing of SHP-1 restored cystine uptake (Figure 3C), which was regulated by system Xc^−^, and attenuated the extent of lipid peroxidation following sorafenib treatment (Figure 3D). In addition, ectopic expression of STAT3 not only restored GSH levels (Figure 4C), but also reduced the burden of lipid ROS associated with sorafenib treatment (Figure 4D), and rescued HCC cells from ferroptotic cell death (Figure 4A). MCL1 is an anti-apoptotic molecule that is transcriptionally regulated by STAT3. Degradation of MCL1 frees BECN1 [23,24], and the dissociated BECN1 subsequently triggers autophagy [30]. Our results showed that by enhancing the activity of SHP-1 (Figure 7C) and dephosphorylating STAT3 (Figure 3A and Figure 4A), sorafenib downregulated the expression of MCL1 (Figure 3A and Figure 4A). Less MCL1 was expressed and engaged with BECN1, thus facilitating the coupling of BECN1 and SLC7A11 (Figure 5A,B), leading to inhibition of system Xc^−^. The declined cystine intake following sorafenib treatment due to the inactivated system Xc^−^ gave rise to less GSH and impaired the function of GPX4. Inhibition of GPX4 then contributed to the accumulation of lipid peroxidation and triggered ferroptosis in HCC cells. Our work reported herein uncovered the mechanism of action (as illustrated in the graphical abstract) by which sorafenib triggers ferroptosis in HCC—via SHP-1/STAT3-regulated MCL1:BECN1 decoupling and the consequent BECN1:SLC7A11 coupling. In addition, our findings revealed an anti-ferroptotic role of MCL1, by competing with SLC7A11 for binding with BECN1 and preventing the assembly of a pro-ferroptotic complex consisting of BECN1 and SLC7A11. We also discovered that BECN1, while known for mediating autophagy, may also promote ferroptosis by dissociating itself from the other BH3 domain-containing molecule MCL1, and instead coupling with SLC7A11.

STAT3 has been correlated with a pro-oncogenic role in HCC, regulating survival, proliferation, immune suppression, and invasion [31]. It has been reported that phosphorylated nuclear STAT3 was detected in approximately 60% of human HCC samples, but not in the adjacent, non-neoplastic liver specimens [32]. Phosphorylation of STAT3, which was linked to greater tumor size and increased recurrence [33], was also elevated in HCC patients with poorer prognosis [34]. TTI-101, a small-molecule STAT3 inhibitor that competitively targets the phosphotyrosyl peptide-binding pocket in the Src homology 2 (SH2) domain of STAT3, has shown anti-HCC activities [35,36] and received orphan drug designation for HCC. STAT3 inhibition by napabucasin suppresses HCC growth, probably by enhancing the “eat me” calreticulin signal, reducing the expression of the “don’t eat me” protein CD47, and triggering immunogenic cell death [37]. Suppressing STAT3 signals may also induce the polarization of anti-tumoral M1 macrophages and reshape the HCC tumor microenvironment [38]. Sorafenib is an approved medication for the treatment of HCC and has been shown to inactivate STAT3 [15,29,39,40,41]. We report herein that ferroptosis is one of the major contributors to sorafenib-induced HCC cell death (Figure 1A–C), and that STAT3 inhibition by sorafenib plays a prominent role—via downregulation of MCL1 expression (Figure 4A) and facilitation of BECN1 binding to SLC7A11 (Figure 5A,B)—in sorafenib-triggered ferroptosis in HCC. The findings of the present study further consolidate the concept of STAT3 as a promising target for the treatment of HCC.

It is also notable that STAT3 can be detected in the mitochondria of the primary liver [42] and may positively regulate the function of complexes I and II in the electron transport chain [42], which generate ROS. Our results showed that STAT3 exerts another approach for modulating cellular ROS levels, by transcriptionally regulating the expression of MCL1. SHP-1 activation and STAT3 dephosphorylation induced by sorafenib reduced the expression of MCL1, reinforcing the BECN1–SLC7A11 interaction, inhibiting system-Xc^−^-mediated antioxidant defense, and contributing to ferroptosis. Our findings also indicate the possibility of using BECN1 mimetics or MCL1 inhibitors to disrupt the BECN1–MCL1 association, leading to inhibition of SLC7A11, which further triggers HCC cell death through ferroptosis. Inhibition of SLC7A11 by sulfasalazine increases ROS levels and suppresses HCC tumor growth [43]. Recently, Iseda et al. reported that lenvatinib, which is another multiple tyrosine kinase inhibitor (TKI) and first-line treatment for HCC, downregulated the expression of SLC7A11 and induced ferroptosis in HCC cells [44]. The findings described above indicate that HCC cells may be susceptible to ferroptotic cell death triggered by the suppression of SLC7A11, the expression of which was correlated with poorer survival of HCC patients (Supplementary Figure S3B).

Interestingly, we observed an increase in SLC7A11 expression following sorafenib treatment (Figure 5A,B) in both Huh7 and Hep3B cells, so we cannot rule out the possibility that the increased interaction between BECN1 and SLC7A11 was partially due to the enhanced expression of SLC7A11. The observation that sorafenib treatment upregulates the expression of SLC7A11 is noteworthy, as most reports indicate that sorafenib inhibits the activity of system Xc^−^ [27,28]. Yuan et al. also reported that sorafenib reduced the expression of SLC7A11 in hepatic stellate cells and alleviated liver fibrosis [45]. On the other hand, it has been suggested that the increased SLC7A11 expression observed after treatment with ferroptosis-inducing agents to inhibit system Xc^−^ may be a negative feedback control to prevent the overaccumulation of lipid peroxidation [4]. Still, the decreased cystine intake following sorafenib treatment observed in our experiments (Figure 2A) suggests that SLC7A11 inhibition due to increased BECN1 binding outweighs the effects of upregulated SLC7A11 expression in sorafenib-triggered HCC ferroptosis.

Considering the lack of actionable driver mutations for HCC, perturbing the redox homeostasis and triggering of ferroptotic cell death may be a promising avenue for the treatment of HCC. Sorafenib has been linked to induction of ferroptosis in HCC, but the detailed molecular interactions mediating sorafenib-induced ferroptosis remain unresolved. Wang et al. previously hinted that glutathione S-transferase zeta 1 (GSTZ1) may enhance ferroptosis associated with sorafenib treatment by suppressing nuclear factor erythroid 2-related factor 2 (NRF2)-regulated GPX4 transcription [46]. A more recent report suggested that LIF receptor subunit alpha (LIFR) sensitizes HCC cells to sorafenib-induced ferroptosis through NF-κB inhibition and the resultant downregulation of iron-sequestering lipocalin 2 (LCN2) [47]. Our data demonstrate that through elevation of SHP-1 activity and inactivation of STAT3, sorafenib reduces the expression of MCL1. MCL1 assumes an insulating role and sequesters BECN1. Downregulation of MCL1 following sorafenib treatment therefore increases the levels of available BECN1 and enables more binding between BECN1 and SLC7A11. This increased association between BECN1 and SLC7A11 leads to the inhibition of system Xc^−^, the accumulation of lipid ROS, and the triggering of ferroptosis in sorafenib-treated HCC cells. Our study outlines the molecular interactions associated with sorafenib-induced ferroptosis in HCC and supports further development of rationally designed ferroptosis-inducing agents to treat HCC.

## 4. Materials and Methods

### 4.1. Reagents, Chemicals, and Antibodies

Sorafenib tosylate (HY-10201A) and RSL3 (HY-100218A) were purchased from MedChemExpress (Monmouth Junction, South Brunswick, NJ, USA). Liprox-1 (T2376) and DFO (T1637) were purchased from TargetMol (Wellesley Hills, Wellesley, MA, USA). For cell-based studies, chemicals were dissolved in dimethyl sulfoxide (DMSO) (D2650) and then added to 10% Dulbecco’s modified Eagle medium (DMEM). 2-ME (M3148), L-glutathione reduced (GSH) (G4251), cystine–FITC (SCT047), and 3-[4,5-dimethylthiazol-2-yl]-2,5-diphenyltetrazolium bromide (MTT) (M2128) were obtained from Sigma-Aldrich (St. Louis, MO, USA). DharmaFECT 4 transfection reagent (T-2004-03), siSHP-1 (PTPN6, L-009778-00-0005), siBECN1 (L-010552-00-0005) and siControl (D-001810-10-50) were obtained from Dharmacon (Lafayette, CO, USA). Human pCMV6-SHP-1-Myc-DDK plasmid (RC213896), pCMV6-STAT3-Myc-DDK plasmid (RC215836), and pCMV6-MCL1-Myc-DDK plasmid (RC200521) were purchased from OriGene (Rockville, MD, USA). Lipofectamine 3000 reagent (L3000-015), C11-BODIPY (D3861), and RediPlate 96 EnzChek Tyrosine Phosphatase Assay kits (R22067) were obtained from Thermo Fisher Scientific (Bridgewater, NJ, USA). The GSH assay kit (ab239709) and the Lipid Peroxidation (MDA) assay kit (ab118970) were obtained from Abcam (Cambridge, MA, USA). The Annexin V/PI assay kit (AVK250) was obtained from Strong Biotech Corporation (Taipei, Taiwan). The antibodies used in the immunoblotting experiments are indicated below. Mouse anti-actin antibodies (66009-1-lg; 1:10,000) were obtained from Proteintech (Rosemont, IL, USA). 4-HNE (ab46545; 1:500) was purchased from Abcam. Goat anti-rabbit IgG-HRP (Santa Cruz Biotechnology Cat# sc-2004; 1:10,000), goat anti-mouse IgG-HRP (Santa Cruz Biotechnology Cat# sc-2005; 1:10,000), normal mouse IgG1 (Santa Cruz Biotechnology Cat# sc-3877; 1 µg for immunoprecipitation), and normal rabbit IgG (Santa Cruz Biotechnology Cat# sc-2027; 1 µg for immunoprecipitation) antibodies were acquired from Santa Cruz Biotechnology (Dallas, TX, USA). Other antibodies, such as p-STAT3 (Y705) (#9145; 1:1000), STAT3 (#9139; 1:3000), MCL1 (#4572 and #94296; 1:1000 for Western blots and 1 µg for immunoprecipitation), SLC7A11 (#12691; 1:1000 for Western blots and 1 µg for immunoprecipitation), BECN1 (#3495 and #4122; 1:1000 for Western blots and 1 µg for immunoprecipitation), Myc-tag (#2276; 1:1000), and tubulin (#2128; 1:1000), were all from Cell Signaling Technology (Danvers, MA, USA). SHP-1 (620302; 1:5000 for Western blots and 1 µg for immunoprecipitation) and goat anti-rat IgG-HRP (112-035-003; 1:10,000) were obtained from BioLegend (San Diego, CA, USA) and Jackson ImmunoResearch (West Baltimore Pike, West Grove, PA, USA), respectively. DMEM (12100-046), Opti-MEM (31985-070), 10× phosphate-buffered saline (PBS) (21600-069), Hanks’ Balanced Salt Solution (HBSS) (14025-076), and 10× trypsin–EDTA (15400-054) were purchased from Thermo Fisher Scientific. Fetal bovine serum (FBS) (04-001-1A) and PSA (BII03-033-1B) (penicillin, streptomycin, and amphotericin B solution) were obtained from Biological Industries (Cromwell, CT, USA).

### 4.2. Cell Culture

Huh7 cells (JCRB0403) were acquired from the Japanese Collection of Research Bioresources (JCRB, Osaka, Japan), while the Hep3B (HB-8064) and HepG2 (HB-8065) cell lines were purchased from the American Type Culture Collection (ATCC, Manassas, VA, USA). Cells were cultured in DMEM supplemented with 10% FBS and 1% PSA in a 37 °C humidified incubator with an atmosphere of 5% CO_2_ in the air. When the cultured HCC cells reached 80% confluence, the cells were washed twice with PBS to remove the FBS. After that, trypsin solution was added to the culture dish and incubated at 37 °C for 5 min to detach the cells. The detached cells were then mixed with an equal volume of FBS-containing DMEM and transferred to a centrifuge tube. Cell pellets were collected by centrifugation at 900 rpm for 5 min. The supernatant was then removed, and the HCC cells were resuspended in fresh medium. The cell suspension was added to a new culture dish at an appropriate spilt ratio (all 1:4–1:6) or was counted with a hemocytometer. The cells were seeded at a density of 1 × 10^4^/cm^2^ and cultured in a CO_2_ incubator.

### 4.3. Flow Cytometry Analysis

The cystine–FITC uptake assay was used to detect the activity of system Xc^−^, while C11-BODIPY was used to determine the production of lipid ROS. In brief, the cells were seeded in 6-well plates (3 × 10^5^ cells/well) and exposed to the indicated drugs. After that, the cells were harvested by trypsinization. The cells were subsequently stained with HBSS containing cystine–FITC (2 µM) or C11-BODIPY (2.5 µM) in a 37 °C culture incubator for 20 min. The cells were next resuspended in 500 µL of fresh HBSS and analyzed using a flow cytometer (CytoFLEX, Beckman Coulter, Brea, CA, USA) equipped with a 488 nm laser for excitation. Data were acquired from the FL1 channel. A minimum of 10,000 cells were analyzed for each condition, and the mean values of fluorescence were quantified using CytExpert 2.4 software (Beckman Coulter, CA, USA).

### 4.4. GSH Assay

The level of total GSH was measured using the Glutathione Assay Kit according to the manufacturer’s instructions. The measurement of total GSH is based on the glutathione recycle system by 5,5′-dithiobis (2-nitrobenzoic acid) (DTNB) and glutathione reductase. In brief, DTNB reacts with GSH and generates 5-thio-2-nitrobenzoic acid (yellow product, O.D. 412 nm). The formed GSSG is then reduced back to GSH by glutathione reductase, and GSH interacts with DTNB again to produce more 5-thio-2-nitrobenzoic acid. Cells were seeded in 6 cm dishes at a density of 6 × 10^5^ cells/dish. After treatment for 16 h, the cells were scraped into ice-cold 1% sulfosalicylic acid/glutathione buffer and lysed via sonication. After centrifugation, 20 µL of the supernatant was blended with 180 µL of assay buffer (containing DTNB, NADPH-generating mix, and glutathione reductase) in a 96-well plate for 5 min at room temperature, followed by absorbance measurement at 412 nm. The values of the treated groups were normalized to that of the DMSO control.

### 4.5. Lipid Peroxidation Assay

The relative level of lipid peroxidation in cell lysates was detected using a Lipid Peroxidation Assay Kit following the manufacturer’s instructions. Briefly, the free MDA in the samples interacts with thiobarbituric acid (TBA) to form the MDA–TBA adduct, which can then be quantified colorimetrically (pink products, O.D. 532 nm). At least 1 × 10^6^ cells per reaction were homogenized by sonication in 150 µL of ddH_2_O and 3 µL of butylated hydroxytoluene. One volume of 2 N perchloric acid was further added to precipitate proteins. Then, the clear supernatant was collected by centrifugation at 13,500 rpm at 4 °C for 10 min, and 200 µL of the supernatant was heated with 600 µL of TBA solution at 95 °C for 1 h, followed by cooling on ice for 10 min. After that, 200 µL of the mixture was pipetted into a 96-well microplate, and the absorbance was determined at 532 nm.

### 4.6. Western Blot Analyses

HCC cells were harvested and collected using a modified radioimmunoprecipitation (RIPA) lysis buffer (150 mM NaCl, 1 mM ethylenediaminetetraacetic acid (EDTA), 0.25% sodium deoxycholate, 1% NP-40, and 50 mM Tris-HCl, pH 7.4) freshly supplemented with 1 mM PMSF, 1× protease inhibitor, 0.1% sodium dodecyl sulfate (SDS), 1 mM NaF, and 1 mM Na_3_VO_4_. The cell lysates were incubated on ice for 30 min before centrifugation at 13,500 rpm for 30 min. The supernatants were then collected for protein quantification. Equal amounts of protein were subjected to SDS–polyacrylamide gel electrophoresis (PAGE) for 120 min at 120 V and then transferred to polyvinylidene difluoride membranes. The membranes were next blocked and immunoblotted with the indicated primary antibodies overnight at 4 °C. The membranes were further probed with secondary antibodies for another 1 h at room temperature. The membranes were next soaked in an enhanced chemiluminescence solution (WBKLS0500) (Millipore, Billerica, MA, USA), and the signals were detected using the UVP ChemiDoc-It 815 Imaging System (Analytik Jena US LLC, Upland, CA, USA). The relative signal intensity of each protein band was normalized to that of the control and was quantitatively shown under each band of interest.

### 4.7. Immunoprecipitation Analysis

Cells were collected and lysed in ice-cold immunoprecipitation (IP) buffer (1 mM EDTA, 50 mM Tris-HCl pH 7.4, 120 mM NaCl, 1% NP-40, and 0.25% sodium deoxycholate). The cell lysates were then collected by centrifugation at 13,500 rpm for 20 min at 4 °C and subjected to protein concentration determination using the bicinchoninic acid reagent (23,225) (Thermo Fisher Scientific, Bridgewater, NJ, USA). Prior to immunoprecipitation, the cell lysates containing equal amounts of protein (0.75–1 mg) were pre-cleaned with protein A agarose beads (16–125) (Millipore, Billerica, MA, USA) for 30 min at 4 °C and, subsequently, incubated with IgG control, anti-SLC7A11, anti-BECN1, or anti-MCL1 antibodies overnight with rotation. The next day, protein A agarose beads were added and incubated at 4 °C for another 3 h. The beads were then washed four times with IP buffer, and the precipitated proteins were eluted by boiling in SDS sample buffer for 10 min before performing SDS–PAGE.

### 4.8. Reverse-Transcription Quantitative Polymerase Chain Reaction (RT-qPCR)

Total RNA was isolated using an RNA extraction kit (AM1912) (Ambion-Invitrogen, Lennik, Belgium). The cDNA was prepared from 2.5 µg of RNA by using the SuperScript Vilo cDNA Synthesis Kit (11754-050) (Invitrogen, Carlsbad, CA, USA) and subjected to real-time PCR with SYBR Green reagents (A25742) (ABI, Carlsbad, CA, USA) following the manufacturer’s instructions. The primer sequences were designed as follows: *MCL1*, forward 5′-CTTGCCACTTGCTTTTCTGG-3′ and reverse 5′-CAAGGCATGCTTCGGAAACT-3′; *GAPDH*, forward 5′-GTCTCCTCTGACTTCAACAGCG-3′ and reverse 5′-ACCACCCTGTTGCTGTAGCCAA-3′. Three samples per condition were analyzed using a Rotor-Gene Q machine (Qiagen, Germantown, MD, USA).

### 4.9. SHP-1 Activity Measurement

After termination of treatment, the nude mice were euthanatized. The tumors were harvested, and immunoprecipitation was performed using antibodies against SHP-1. A RIPA buffer with protease inhibitors was used for cell lysis. Then, protein quantification was performed, and 750–1000 µg of protein was incubated with 1 µg of antibodies against SHP-1 at 4 °C overnight. After that, protein G beads (17-0618-01) (GE Healthcare Bio-Sciences, Piscataway, NJ, USA) were added and reacted for 3 h. The beads were further washed with RIPA buffer before the SHP-1-containing cell lysates were collected. The cellular SHP-1 activity was further assessed using the RediPlate 96 EnzChek Tyrosine Phosphatase Assay Kit. Fluorescence was measured at 452 nm.

### 4.10. Xenograft Tumor Model

Male nude mice (4 weeks old) were acquired from the National Laboratory Animal Center (Taipei, Taiwan). All experimental procedures using these mice were conducted following the protocol (code: IACUC-110C-003) approved by the Institutional Laboratory Animal Care and Use Committee of Cardinal Tien Hospital on 03/Feb/2021. Each nude mouse was inoculated subcutaneously at the dorsal flank with 5 × 10^6^ Huh7 cells suspended in 0.1 mL of PBS containing 50% Matrigel (356,235) (BD Biosciences, Bedford, MA, USA). After the tumors reached 100–200 mm^3^, the mice were treated with sorafenib (15 mg/kg) or vehicle (ethanol/Cremophor EL/water = 1:1:6) per os (p.o.) once daily. The mice’s body weights and tumor volumes were measured and recorded twice weekly.

### 4.11. Statistics

Values are shown as the mean ± standard deviation (SD). Statistics were analyzed by employing the Mann–Whitney U test or two-way analysis of variance (ANOVA) using GraphPad Prism version 6.0 (GraphPad Software, San Diego, CA, USA); *p* < 0.05 was considered to be statistically significant.

## Data Availability

The data reported in the current study are available from the corresponding author upon request. Further information regarding the materials and methods are available in the Supplementary Materials section.

## Supplementary Materials

The following supporting information can be downloaded at: https://www.mdpi.com/article/10.3390/ijms231911092/s1.
