# Supplementary material for: SHP-1/STAT3-Signaling-Axis-Regulated Coupling between BECN1 and SLC7A11 Contributes to Sorafenib-Induced Ferroptosis in Hepatocellular Carcinoma
Source: Int J Mol Sci. 2022 Sep 21;23(19):11092. doi: 10.3390/ijms231911092 (PMC9570040; doi:10.3390/ijms231911092)
Supplement: Supplementary file 1 [file ijms-23-11092-s001.zip › ijms-1907698-supplementary.pdf]

SHP-1/STAT3-Signaling-Axis-Regulated Coupling between BECN1 and SLC7A11 Contributes to Sorafenib-Induced Ferroptosis in Hepatocellular Carcinoma

Supplementary Materials and Methods

Cell death analysis

After treatment for 16 hours, the Annexin V/PI assay (Strong Biotech Corporation, Taipei, Taiwan) was performed for cell death analysis. Briefly, fresh cells were washed once in PBS before incubation with annexin v-FITC in a binding buffer containing PI for 10 minutes in the dark. After incubation, 0.4 ml binding buffer was added to resuspend the cells. The solution was then transferred to FACS tubes and analyzed using a flow cytometer (CytoFLEX, Beckman Coulter, Brea, CA, USA). A minimum of 10,000 cells were counted and analyzed per condition.

Plasmid transfection

3-5 μg of pCMV6-SHP-1-Myc-DDK plasmid, pCMV6-STAT3-Myc-DDK plasmid, pCMV6-MCL1-Myc-DDK plasmid or pCMV6-Entry-vector was used for transient transfection. Each group of plasmids was mixed with 6-10 μl P3000 reagent and 250 μl opti-MEM in tube 1. Then, 9-15 μl of Lipofectamine 3000 was incubated with 250 μl opti-MEM in tube 2. After that, the two tubes were mixed and incubated for 15 minutes. Next, the mixture was added into the cells in a 10-cm culture dish for 24 hours at 37°C. The transfected cells were then washed prior to further experiments.

siRNA transfection

20 μl of siRNA was incubated with 230 μl of opti-MEM in tube 1 for 5 minutes. 16 μl of DharmaFECT 4 transfection reagent and 234 μl of opti-MEM were incubated in tube 2 for 5 minutes. Then, tube 1 and tube 2 were gently mixed and incubated for 15 minutes.

The mixture was then added to a well containing 3,500 µl of cell culture medium in a 10-cm dish. The final concentration of siRNA in each well was 100 nM. The 10-cm dish was placed in a 37°C CO2 incubator for 24 hours before further experiments were conducted.

Cell viability

HCC cells were seeded at a density of 3000-4000 cells/well in a 96-well plate. Following attachment, the cells were incubated with the indicated chemicals. After that, 25 µl of 5X MTT solution was added to the wells to reach a final concentration of MTT at 1 mg/ml. The 96-well plate was then incubated in a CO2 incubator for 3 hours. Next, the supernatant was removed and 100 µl of DMSO was added to solubilize the purple MTT crystals. The number of viable cells per well was directly proportional to the intensity of the formazan dye (purple), and was assessed by measuring the absorbance at 570 nm using a microplate reader (BioTeK Synergy HT, Winooski, VT, USA).

SLC7A11 expression analysis

The tumor immune estimation resource (TIMER) on-line database (https://cistrome.shinyapps.io/timer/) was utilized to compare the SLC7A11 expression between HCC tumors and their surrounding normal tissues. The Kaplan-Meier survival curves were plotted using the "Survival" module in the TIMER database to examine whether high (top 50%) or low (bottom 50%) SLC7A11 expression correlates with HCC patient' survival.

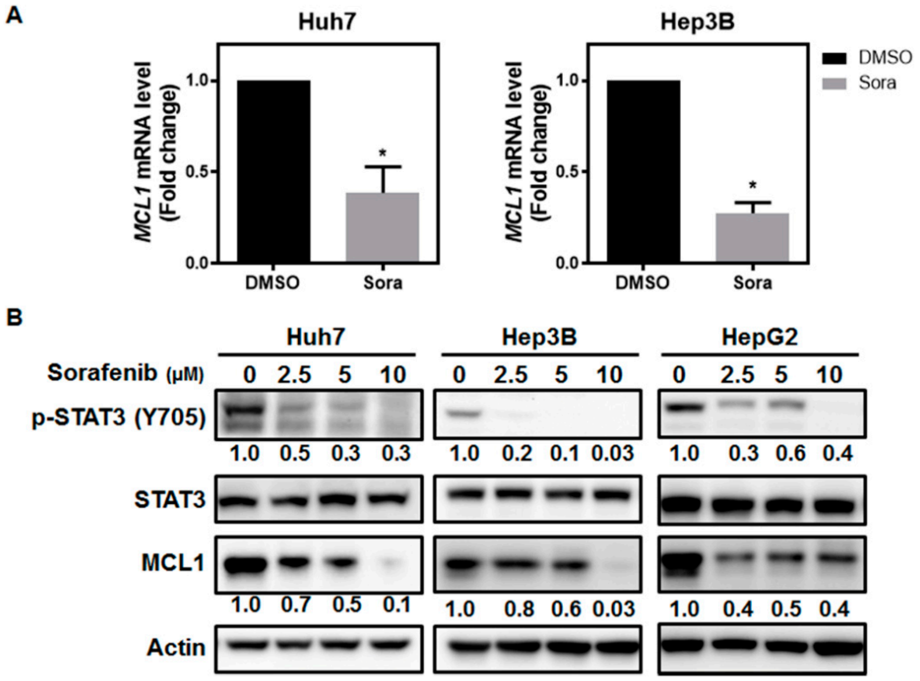

Supplementary Figure S1. Sorafenib reduces MCL1 expression in HCC cells. (A) Decreased expression of *MCL1* mRNA was observed following sorafenib treatment. Huh7 cells and Hep3B cells were treated with 2.5 μM and 5 μM of sorafenib for 16 hours, respectively. The mRNA levels of *MCL1* were determined by RT-qPCR. (B) A dose-dependent reduction of MCL1 proteins were recorded in HCC cells treated with sorafenib. Columns mean; bars, SD ($n \geq 3$). Statistical significance was evaluated by using Mann-Whitney U test. (*, $p < 0.05$).

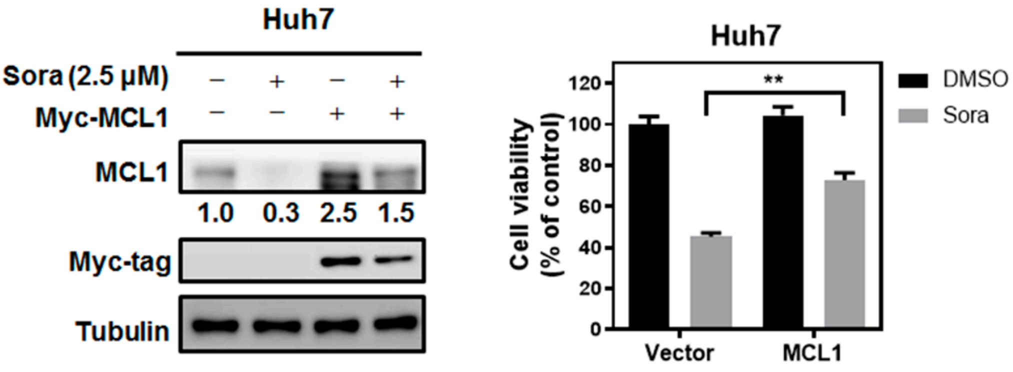

Supplementary Figure S2. Ectopic expression of MCL1 protects HCC cells from sorafenib-induced ferroptotic cell death. MTT assays were conducted to analyze cell viability in HCC cells with ectopically expressed MCL1 after sorafenib treatment. Columns mean; bars, SD ($n \geq 3$). Statistical significance was analyzed by Mann-Whitney U test. (**, $p < 0.01$).

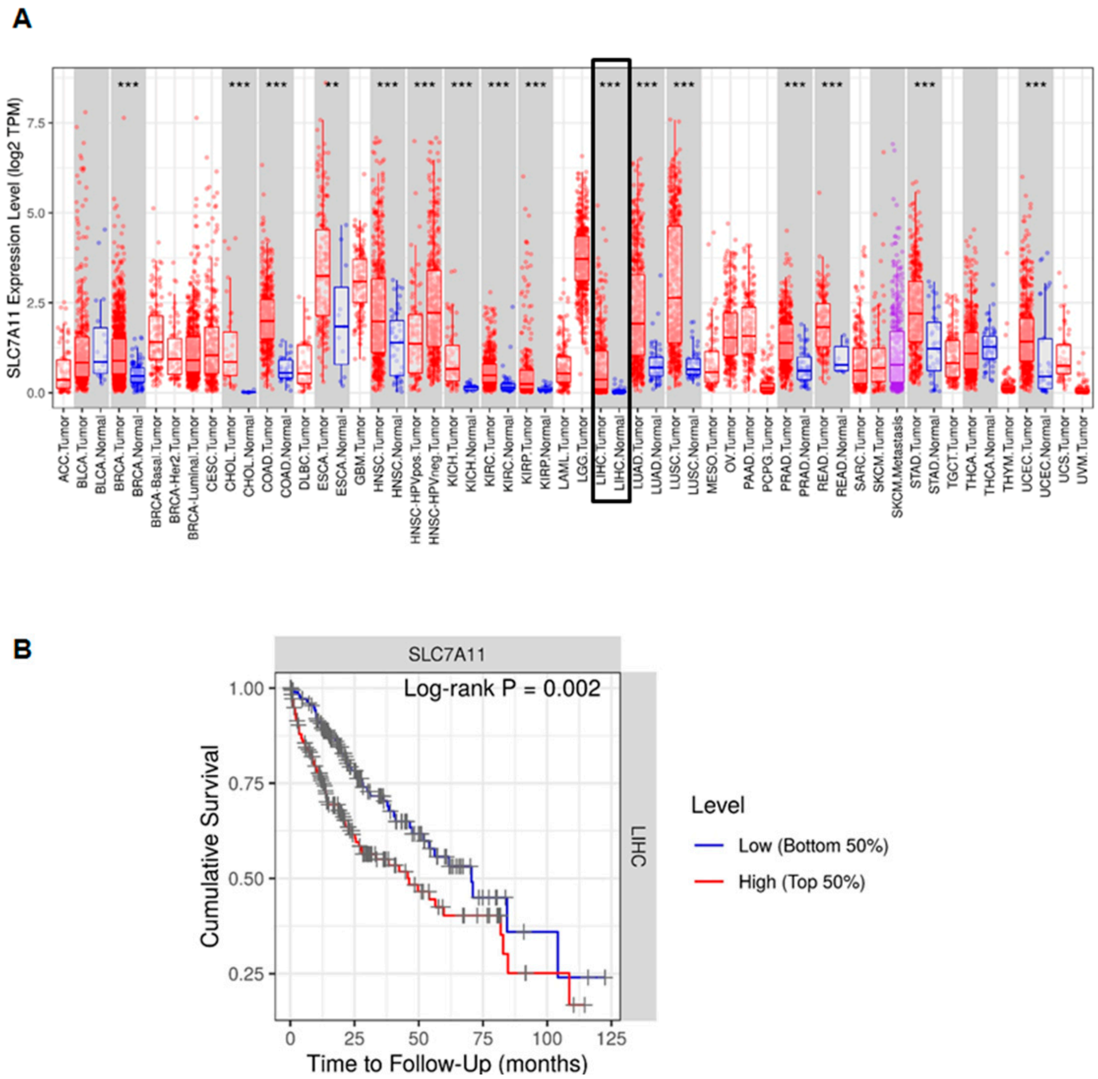

Supplementary Figure S3. The expression of SLC7A11 in HCC is associated with poor outcomes. (A) The differential expression of SLC7A11 between cancers and their adjacent normal tissues across different TCGA tumors in the TIMER database. Statistical significance was evaluated by Wilcoxon test. (**, $p < 0.01$; ***, $p < 0.001$). (B) Kaplan-Meier survival curves showing the correlation between SLC7A11 expression and HCC patient' survival. Data was analyzed by employing the TIMER database.
